# Disinfectants

**Published:** 1886-04

**Authors:** 


### DISINFECTANTS.

Are those deodorisers which, in purifying the air, prevent the spread of contagious diseases. The following are several:

1. Sir William Burnett's is a concentrated solution of chloride of zinc, supplied in a fluid state.

2. Collins's is chloride of lime, 2 parts; burnt alum, 1. Used either dry or strongly infused in water.

3. Ellerman's is obtained from chiorides of iron and manganese. It is strongly recommended for the deodorisation of sewage.

4. Condy's is a preparation of alkalies and manganese.

5. Water, 8 parts; nitrate of lead, 1 part.

6. Sulphate of lime, 53 parts; sulphate of iron, 40; sulphate of zinc, 7; pear chercoal.

7. Water, 6 pints; nitrate acid, 12 oz; litharge, 13½ oz.

8. Sulphate of iron, 20 parts; sulphate of zinc, 10; powdered tan, 4: tar, 1; oil, 1; strongly recommended for cess-pools.

